# Transport properties of two-dimensional dissipative flow of hybrid nanofluid with Joule heating and thermal radiation

**DOI:** 10.1038/s41598-022-23337-z

**Published:** 2022-11-12

**Authors:** Aisha M. Alqahtani, Maawiya Ould Sidi, M. Riaz Khan, Mohamed Abdelghany Elkotb, Elsayed Tag-Eldin, Ahmed M. Galal

**Affiliations:** 1 Department of Mathematical Sciences, College of Science, Princess Nourah bint Abdulrahman University, P. O. Box 84428, Riyadh, 11671 Saudi Arabia; 2 RT-M2A Laboratory, Mathematics Department, College of Science, Jouf University, P.O. Box: 2014, Sakaka, Saudi Arabia; 3 Department of Mathematics, Quaid-i-Azam University, 45320, Islamabad, 44000 Pakistan; 4 Mechanical Engineering Department, College of Engineering, King Khalid University, Abha, 61421 Saudi Arabia; 5 Mechanical Engineering Department, College of Engineering, Kafrelsheikh University, Kafr el-Sheikh, 33516 Egypt; 6 Faculty of Engineering and Technology, Future University in Egypt, New Cairo, 11835 Egypt; 7 Department of Mechanical Engineering, College of Engineering in Wadi Alddawasir, Prince Sattam bin Abdulaziz University, Wadi Alddawasir, Saudi Arabia; 8 Production Engineering and Mechanical Design Department, Faculty of Engineering, Mansoura University, P.O 35516, Mansoura, Egypt

**Keywords:** Applied mathematics, Software, Fluid dynamics

## Abstract

The important feature of the current work is to consider the pressure variation, heat transport, and friction drag in the hydromagnetic radiative two-dimensional flow of a hybrid nanofluid depending on the viscous dissipation and Joule heating across a curved surface. The curved surface has been considered with the binary heating process called as prescribed heat flux and surface temperature. The basic partial differential equation (PDEs) has been converted into the non-dimensional ordinary differential equations (ODEs) by applying some specified dimensionless transformations. The bvp4c built-in package in MATLAB has been considered to find the numerical solution of the consequential equations. The graphical results have been plotted in terms of pressure, friction drag, velocity, temperature, and heat transport. Several important results have also been plotted for the plan level surface $($The condition of $K\to \infty )$. It is found that the heat transport rate respectively reduces and enhances with the enhancement of radiation parameter and Hartmann number as well as the friction drag is enhancing with the high-volume fraction of nanoparticles and Hartmann number. Moreover, enhancing curvature parameter, enhances the friction drag and declines the heat transport rate. The current work renders uncountable applications in several engineering and industrial systems like electronic bulbs, electric ovens, geysers, soil pollution, electric kettle, fibrous insulation, etc. Moreover, the heating as well as the cooling systems of electrical, digital, and industrial instruments, are controlled by the heat transport in fluids. Thus, it is important to use such flows in these types of instruments.

## Introduction

Scientists are seriously interested in the study of nanofluids considering their abilities to transport a higher rate of heat used in applications of various industries. The ability of heat transportation of the nanofluids has been recently rectified through the distributions of two different kinds of nanoparticles in the base liquids. Some of the interesting studies on nanofluids are^1–3^. This modern type of fluid is characterized as a hybrid nanofluid which renders greater transport of heat as compared with nanofluids. Several engineering, as well as industrial applications, can be operated with a hybrid nanofluid like cooling of the atomic system, solar water heating, electronic cooling, heat exchanger, drug reduction, microchannel, refrigerators, generator cooling, transformers, heating and cooling in buildings, vehicle brake fluids, lubricants, grinding, heat pipes, etc. The approach of hybrid nanomaterials is significant in modern areas of research like biology, engineering, agriculture, and applied sciences. The rectified thermal and physical features are associated with the classification of innovative nanomaterials like hybrid nanofluids. The stagnation point flow with the existence of dual solutions based on the hybrid nanofluid and curved surface has been studied by Khan et al.^4^. The approach of the computational model towards the convective rotating surface depending on the hybrid nanofluid has been discussed by Hussain et al.^5^. Khan et al.^6^ studied the magnetized flow of different hybrid nanofluids assuming the comparative assessment of friction drag and heat transport including thermal radiation. Ramzan et al.^7^ dealt with the analysis of the impact of nanoparticle shape on the hydrothermal properties of hybrid nanofluid. Redouane et al.^8^ studied the flow of silver and magnesium oxide mixed hybrid nanofluid flow in a hot trigonal enclosure having a cylindrical cavity. Alhowaity et al.^9^ incorporated the power law nanofluid model to examine the thermal features of hybrid nanofluid flow in over a heated surface. Khan et al.^10^ studied the application of hybrid nanofluid in the drilling process where the heat is continuously dissipated.

The heat emission in processes of engineering where a large temperature is desired through the radiation influence is a critical phenomenon in the transportation of heat. The installment of the thermal system, as well as the transport of heat are directly controlled through this phenomenon. Radiation is the speedy process of thermal motion where transport of heat takes place as electromagnetic waves with no reliance on a medium. The influence of thermal radiation is highly significant during the assessment of heat effects based on the high-temperature flow process, particularly in the atomic plants, missiles, turbines, satellites, different propulsion instruments for spacecraft, and the design of reliable devices, and several modern conversions methods. The transport of heat in the manufacturing of polymer is also controlled through thermal radiation. Zhou et al.^11^ studied the thermal radiation effect in a time-dependent flow of magnetized Casson fluid including the influence of heat source across a stretched porous surface. Liu et al.^12^ extended the work of a time-dependent Casson fluid towards the stretched convective surface and discussed the thermal effects of radiation and magnetic field. Khan et al.^13^ determined the heat transfer based on the approach of the computational model towards the Casson fluid depending on the magnetic field, thermal radiation, and stretching/shrinking sheet. Moreover, the work of Elkotb et al.^14^ addresses the chemically reactive flow of magnetized nanofluid including the effect of thermal radiation, viscous dissipation, and heat generation absorption over the heat and mass transport. Puneeth et al.^15,16^ studied the characteristics of jet flow of Casson nanofluid under the influence of microorganisms. Bilal et al.^17^ discussed the impact of the prominent thermal radiation parameter on the heat transfer properties of a nanofluid flowing past a porous stretching sheet. Sullivan et al.^18^ implemented deep learning algorithm to study the thermal radiation impact on the hybrid nanofluid. Additional studies with the consideration of thermal radiation can be seen in the refs.^19–22^.

The flow of electric current across an electrical conductor causes to produce thermal energy which boosts the material temperature of the conductor and produces heat known as Ohmic or Joule heating. The flow of the magnetized fluid is very sensitive to the phenomenon of Joule heating. The majority of the electric and electronic instruments are practically running using the Joule heating effects. Khan et al.^23^ observed that the heat generation/absorption and the Joule heating effects control the entropy generation and heat transportation in Darcy–Forchheimer flow associated with a stretched nonlinear surface. Zhang et al.^24^ studied the impact of ohmic heating through the stagnation flow of nanofluid including the influence of magnetic field along a convective stretching/shrinking sheet. The two-dimensional dissipative flow of nanofluid coupled with an aligned magnetic field numerically discusses the friction drag and heat transport connected to the impact of Joule heating and heat generation/absorption^25^. Khan et al.^26^ discussed the magnetized incompressible flow of copper–water nanofluid investigating the advantages of Joule heating, mass suction, and viscous dissipation over the skin friction and heat transportation.

The action of shear stress in the flow system allows extra heat which is described as viscous dissipation. It appears in heavy gases, massive planets, powerful gravitational fields, and geographical systems. Moreover, it is highly considered in the assessment of heat transport, especially in the boundary layer motion of fluid due to the high-velocity gradients. The temperature reduction appears as a heat source giving rise to a significant increase in fluid temperature. The higher gradients of velocity inside the boundary layer flow trigger the transformation of the kinetic energy of the fluid into heat energy and as a result, the fluid temperature enhances. Khan et al.^26^ discussed the viscous dissipation influence over the magnetized two-dimensional boundary layer flow of incompressible nanofluid dealing with the association of mass suction and surface convection across a stretched surface. The consideration of the dissipation effect with the two-dimensional power-law flow of incompressible laminar fluid including the slip condition of power-law velocity along a stretched exponential surface was studied by He et al.^27^. In this study, the heat and mass transport behavior were investigated on the bases of Rosseland’s diffusion model including the additional effects of thermal radiation, Hall current, and transverse magnetic field. Haung et al.^28^ investigated the numerical study of viscous nanofluid including the influence of viscous dissipation over the rate of heat transport and friction drag. Additionally, the impact of thermal radiation, magnetic field, and Joule heating was discussed along a stretched curved surface associated with binary heating processes. Khan et al.^25^ determined the heat transport rate based on the approach of the numerical model of incompressible viscous flow of radiative nanofluid depending on the viscous dissipation, aligned magnetic field, heat source/sink, and Joule heating along a convective permeable stretching surface. Additional studies with the consideration of viscous dissipation can be seen in the refs.^29–33^.

The current work determines the influence of Joule heating, thermal radiation, and viscous dissipation over a two-dimensional hydromagnetic flow of a viscous hybrid nanofluid across a stretched curved surface. Additionally, the study also includes heat flux, thermal radiation, and surface temperature. The curved surface has been considered with the binary heating process introduced as surface temperature and prescribed heat flux. The basic PDEs have been transformed into dimensionless ODEs using some specified non-dimensional transformations. The MATLAB built-in package bvp4c has been considered to find the numerical solution to the consequential equations. The diverse values of the dimensionless parameters are used to find the numerical solution which describes the flow characteristics and presents a physical insight of the current work. The stated conditions associated with the stagnation point flow across a curved surface have not been considered in the prior studies. This motivated the authors to consider the existing novel work which numerically describes the heat transportation and friction drag. According to the knowledge of the authors and on the basis of literature survey, the current work is completely different from the existing published articles due to the geometry and associated conditions. The current work renders applications in several engineering and industrial systems like electronic bulbs, electric ovens, geysers, soil pollution, electric kettle, fibrous insulation, etc. Moreover, the heating as well as the cooling systems of electrical, digital, and industrial instruments, are controlled by the heat transport in fluids. Thus, it is important to use such flows in these types of instruments.

From the details of the literature procured, it is noticed that various investigations are performed to explore the fluid motion only for conventional liquids that skip the innovative heat transfer in ethylene glycol (${C}_{2}{H}_{6}{O}_{2}$) comprising alumina ($A{l}_{2}{O}_{3}$) and cupper $(Cu$) nanomaterials. Thus, the research is directed to deal with this novel research gap applicable to the heat transfer enhancement of nanofluids. Therefore, the heat transfer model of a nanofluid is formulated across an infinite semi-region and numerically addressed. The outcomes are acquired towards the flow parameters and considerably examined through computer generated diagrams. Some of the research questions that the article aims are:

- What is the most appropriate model to describe the heat transfer of hybrid nanofluid flowing past a curved sheet?
- How does the radiation and viscous dissipation influence the thermal features of the hybrid nanofluid?
- What is the significance of the volume fraction of suspended nanoparticles in enhancing the thermal properties of the hybrid nanofluid?

## Basic equations

**Figure 1:**
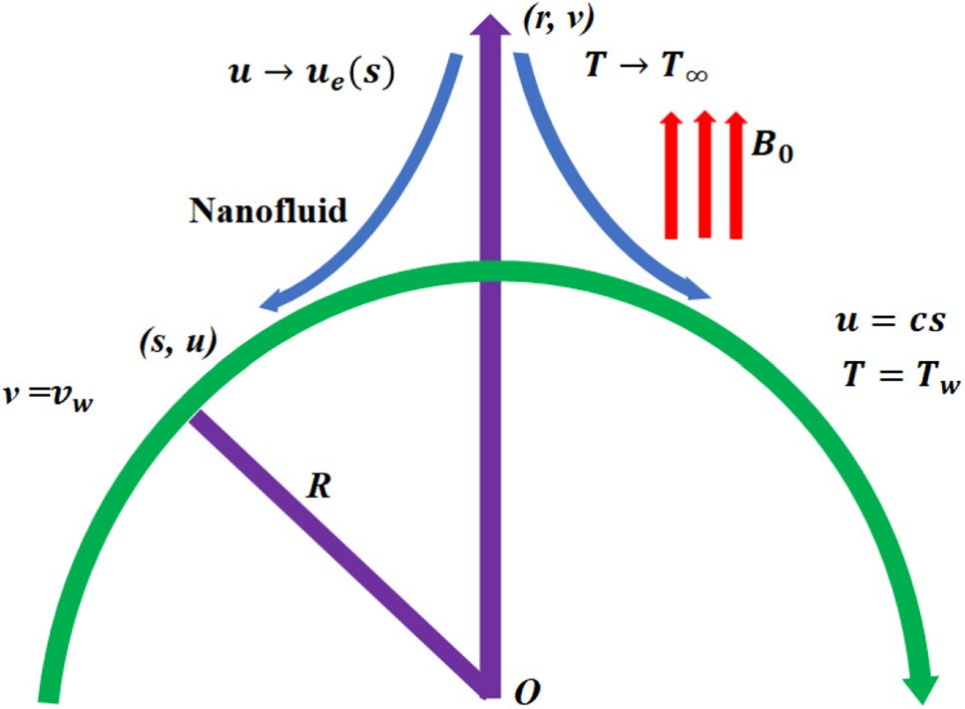
Geometry of the problem.

The two-dimensional magnetized flow of viscous hybrid nanofluid is considered along a curved surface having a radius $$R$$ and depending on the Joule heating and viscous dissipation as shown in Fig. 1. Additionally, the flow is reliant on the thermal radiation, surface temperature $$({T}_{w})$$ and heat flux $$({q}_{w })$$ as well as the hybrid nanofluid is consisting of alumina ($$A{l}_{2}{O}_{3}$$) and cupper $$(Cu)$$ nanoparticles associated with base fluid ethylene glycol ($${C}_{2}{H}_{6}{O}_{2}$$). The ambient temperature is taken to be $${T}_{\infty }$$, where as the stretching and the far field velocity are respectively taken to be $$u=cs$$, and $$u\to {u}_{e}\left(s\right)=as$$. The flow is directed along coordinates $$s$$ and the magnetic flux ($${B}_{0})$$ is considered along coordinates $$r$$ which is taken in the normal direction of the tangential vector.

The governing boundary layer equations and the associated boundary conditions are described in the light of said assumptions as given below^34–36^.

1 $$\frac{\partial }{\partial r}\left[(r+R)v\right]=-R\frac{\partial u}{\partial s},$$

2 $$\frac{1}{{\rho }_{hnf}}\frac{\partial p}{\partial r}-\frac{{u}^{2}}{r+R}=0,$$

3 $$\frac{1}{{\rho }_{hnf}}\frac{R}{r+R}\frac{\partial p}{\partial s}={v}_{hnf}\left(\frac{{\partial }^{2}u}{\partial {r}^{2}}+\frac{1}{r+R}\frac{\partial u}{\partial r}-\frac{u}{{\left(r+R\right)}^{2}}\right)-v\frac{\partial u}{\partial r}-\frac{Ru}{r+R}\frac{\partial u}{\partial s}-\frac{uv}{r+R}+\frac{{\sigma }_{nhf}{{B}_{0}}^{2}}{{\rho }_{hnf}}u,$$

4 $$\frac{{k}_{hnf}}{{\left(\rho {C}_{p}\right)}_{hnf}}\left(\frac{{\partial }^{2}T}{\partial {r}^{2}}+\frac{1}{r+R}\frac{\partial T}{\partial r}\right)-\left(v\frac{\partial T}{\partial r}+\frac{Ru}{r+R}\frac{\partial T}{\partial s}\right)+\frac{{\sigma }_{hnf}}{{\left(\rho {C}_{p}\right)}_{hnf}}{{B}_{0}}^{2}{u}^{2}+\frac{{\mu }_{nf}}{{\left(\rho {C}_{p}\right)}_{hnf}}{\left(\frac{\partial u}{\partial r}-\frac{u}{r+R}\right)}^{2}-\frac{1}{r+R}\frac{\partial {q}_{r}}{\partial r}=0.$$

The term ${q}_{r}$ in Eq. (4) refers to the radiative heat flux. In view of the Rosseland’s relation, this term can be defined as^37,38^.

5 $${q}_{r}=\frac{-4{\sigma }^{*}}{3{k}^{*}}\frac{\partial {T}^{4}}{\partial r}$$

In Eq. (5), the nonlinear term ${T}^{4}$ can be simplified by applying the Taylor series expansion about ${T}_{\infty }$. Assuming the elimination of higher order terms, we can get the following equation.

6 $${T}^{4}\approxeq 4{T}_{\infty }^{3}T-3{\sigma }^{*}{T}_{\infty }^{4}$$

The association of Eq. (6) and Eq. (5) leads to the following equation.

7 $${q}_{r}=\frac{-16{\sigma }^{*}{T}_{\infty }^{3}}{3{k}^{*}}\frac{\partial T}{\partial r}$$

In Eq. (4), replace the values of ${q}_{r}$ presented in Eq. (7) results in the following equation.

8 $$\frac{{k}_{hnf}}{{\left(\rho {C}_{p}\right)}_{hnf}}\left(\frac{{\partial }^{2}T}{\partial {r}^{2}}+\frac{1}{r+R}\frac{\partial T}{\partial r}\right)-\left(v\frac{\partial T}{\partial r}+\frac{Ru}{r+R}\frac{\partial T}{\partial s}\right)+\frac{1}{r+R}\frac{16{\sigma }^{*}{T}_{\infty }^{3}}{3{k}^{*}}\frac{{\partial }^{2}T}{\partial {r}^{2}}+\frac{{\mu }_{hnf}}{{\left(\rho {C}_{p}\right)}_{hnf}}{\left(\frac{\partial u}{\partial r}-\frac{u}{r+R}\right)}^{2}+\frac{{\sigma }_{hnf}}{{\left(\rho {C}_{p}\right)}_{hnf}}{{B}_{0}}^{2}{u}^{2}=0.$$

The following Eqs. (9–11) represents the associated boundary conditions to Eqs. (1–3, 8).

9 $$\left.\begin{array}{c}u=cs,v={v}_{w}=0, \,\,at\,\, r=0,\\ u\to {u}_{e}\left(s\right)=as, \frac{\partial u}{\partial r}\to 0, \,\,as\,\, r\to \infty .\end{array}\right\}$$

### I. Prescribed surface temperature (PST)

10 $$T={T}_{w} \,\,at \,\,r=0\,\,\mathrm{and}\,\, T\to {T}_{\infty } \,\,as\,\, r\to \infty$$

### II. Prescribed heat flux (PHF)

11 $$-k\frac{\partial T}{\partial r}={q}_{w }=D{\left(\frac{s}{l}\right)}^{2}\,\, at\,\, r=0, \,\,and\,\, T\to {T}_{\infty } \,\,as\,\, r\to \infty$$

The boundary conditions in above two Eqs. (10) and (11) refers to the binary heating processes, where ${{T}_{w}>T}_{\infty }$ and $D$ is constant.

Table 1 specifies the thermophysical characteristics of the hybrid nanofluid.

**Table 1:** Mathematical formulations of the thermophysical properties of ${C}_{2}{H}_{6}{O}_{2}-A{l}_{2}{O}_{3}$ and $Cu-{Al}_{2}{O}_{3}/{C}_{2}{H}_{6}{O}_{2}$^39^.

Properties	Nanofluid (${Al}_{2}{O}_{3}-{H}_{2}O$)	Hybrid nanofluid ($Cu-{Al}_{2}{O}_{3}/{H}_{2}O$)
Viscosity	${\mu }_{nf}=\frac{{\mu }_{f}}{{\left(1-\varphi \right)}^{2.5}}.$	${\mu }_{hnf}=\frac{{\mu }_{f}}{{{\left(1-{\phi }_{1}\right)}^{2.5}\left(1-{\phi }_{2}\right)}^{2.5}}$*,*
Electrical conductivity	$\frac{{\sigma }_{nf}}{{\sigma }_{f}}=1+\frac{3\left(\sigma -1\right)\phi }{\left(\sigma +2\right)-\left(\sigma -1\right)\phi }$,	$\frac{{\sigma }_{hnf}}{{\sigma }_{bf}}=\frac{{\sigma }_{{s}_{2}}+2{\sigma }_{bf}-2{\phi }_{2}\left({\sigma }_{bf}-{\sigma }_{{s}_{2}}\right)}{{\sigma }_{{s}_{2}}+2{\sigma }_{bf}+{\phi }_{2}\left({\sigma }_{bf}-{\sigma }_{{s}_{2}}\right)}$*,* where, $\frac{{\sigma }_{bf}}{{\sigma }_{f}}=\frac{{\sigma }_{{s}_{1}}+2{\sigma }_{f}-2{\phi }_{1}\left({\sigma }_{f}-{\sigma }_{{s}_{1}}\right)}{{\sigma }_{{s}_{1}}+2{\sigma }_{f}+{\phi }_{1}\left({\sigma }_{f}-{\sigma }_{{s}_{1}}\right)}$*,*
Heat capacity	${\left(\rho {C}_{p}\right)}_{nf}=\varphi {\left(\rho {C}_{p}\right)}_{s}+\left(1-\varphi \right){\left(\rho {C}_{p}\right)}_{f}.$	${\left(\rho {C}_{p}\right)}_{hnf}={\left(\rho {C}_{p}\right)}_{f}\left(1-{\phi }_{2}\right)\left\{1-{\phi }_{1}+{\phi }_{1}{\left(\rho {C}_{p}\right)}_{{s}_{1}}\right\}+{\phi }_{2}{\left(\rho {C}_{p}\right)}_{{s}_{2}}$
Density	${\rho }_{nf}=\varphi {\rho }_{s}+\left(1-\varphi \right){\rho }_{f}$	${\rho }_{hnf}={\rho }_{f}\left(1-{\phi }_{2}\right)\left\{1-{\phi }_{1}+{\phi }_{1}{\rho }_{{s}_{1}}\right\}+{\phi }_{2}{\rho }_{{s}_{2}}$,
Thermal conductivity	$\frac{{k}_{nf}}{{k}_{f}}=\frac{\left(\frac{{k}_{s}}{{k}_{f}}+2\right)-2\varphi \left(1-\frac{{k}_{s}}{{k}_{f}}\right)}{\left(\frac{{k}_{s}}{{k}_{f}}+2\right)+\varphi \left(1-\frac{{k}_{s}}{{k}_{f}}\right)}.$	$\frac{{k}_{hnf}}{{k}_{bf}}=\frac{{k}_{{s}_{2}}+\left(n-1\right){k}_{bf}-\left(n-1\right){\phi }_{2}\left({k}_{bf}-{k}_{{s}_{2}}\right)}{{k}_{{s}_{2}}+\left(n-1\right){k}_{bf}+{\phi }_{2}\left({k}_{bf}-{k}_{{s}_{2}}\right)}$*,* Where, $\frac{{k}_{bf}}{{k}_{f}}=\frac{{k}_{{s}_{1}}+\left(n-1\right){k}_{f}-\left(n-1\right){\phi }_{1}\left({k}_{f}-{k}_{{s}_{1}}\right)}{{k}_{{s}_{1}}+\left(n-1\right){k}_{f}+{\phi }_{1}\left({k}_{f}-{k}_{{s}_{1}}\right)}$*,*

With the help of following similarity transformations^40^, the governing equations and the boundary conditions can be converted to the dimensionless form.

12 $$\left.\begin{array}{c}u={u}_{e}{f}{^{\prime}}\left(\eta \right)=as{f}{^{\prime}}\left(\eta \right), v=-\frac{R}{r+R}\sqrt{\frac{{{v}_{f}u}_{e}}{s}}f\left(\eta \right)=-\frac{R}{r+R}\sqrt{{av}_{f}}f\left(\eta \right), \\ \eta =\sqrt{\frac{{u}_{e}}{{sv}_{f}}}r=\sqrt{\frac{a}{{v}_{f}}}r, p={\rho }_{f}{{u}_{e}}^{2}P\left(\eta \right), \\ PST: \theta \left(\eta \right)=\frac{T-{T}_{\infty }}{{T}_{w}-{T}_{\infty }}, PHF: T={T}_{\infty }+ \frac{D}{k}{\left(\frac{s}{l}\right)}^{2}\sqrt{\frac{{v}_{f}}{a}}g\left(\eta \right).\end{array}\right\}.$$

Considering the above transformations, Eq. (1) is satisfied automatically, though Eqs. (2, 3, 8) and Eqs. (9–11) leads to the subsequent equations.

13 $$\frac{{\rho }_{f}}{{\rho }_{hnf}}\frac{\partial P}{\partial \eta }=\frac{1}{\eta +K}{f{^{\prime}}}^{2},$$

14 $$\frac{{\rho }_{f}}{{\rho }_{hnf}}\frac{2K}{\eta +K}P=\frac{{v}_{hnf}}{{v}_{f}}\left(f{^{\prime}}{^{\prime}}{^{\prime}}-\frac{1}{{\left(\eta +K\right)}^{2}}f{^{\prime}}+\frac{1}{\eta +K}f{^{\prime}}{^{\prime}}\right)-\frac{K}{\eta +K}{\left({f}{^{\prime}}\right)}^{2}+\frac{K}{\eta +K}f{f}^{{^{\prime}}{^{\prime}}}+\frac{K}{{\left(\eta +K\right)}^{2}}f{f}{^{\prime}}-{M}^{2}\frac{{\sigma }_{hnf}}{{\sigma }_{f}}\frac{{\rho }_{f}}{{\rho }_{hnf}}{f}{^{\prime}},$$

15 $$\frac{1}{Pr}\frac{{k}_{hnf}}{{k}_{f}}\frac{{\left(\rho {C}_{p}\right)}_{f}}{{\left(\rho {C}_{p}\right)}_{hnf}}(1+\frac{4}{3}Rd)\left(\theta {^{\prime}}{^{\prime}}+\frac{1}{\eta +K}\theta {^{\prime}}\right)+\frac{K}{\eta +K}f{\theta }{^{\prime}}+{\frac{{\mu }_{hnf}}{{\mu }_{f}}\frac{{\left(\rho {C}_{p}\right)}_{f}}{{\left(\rho {C}_{p}\right)}_{hnf}}E}_{c}{\left({f}^{{^{\prime}}{^{\prime}}}-\frac{{f}{^{\prime}}}{\eta +K}\right)}^{2}\quad+{{M}^{2}E}_{c}\frac{{\left(\rho {C}_{p}\right)}_{f}}{{\left(\rho {C}_{p}\right)}_{hnf}}\frac{{\sigma }_{hnf}}{{\sigma }_{f}}{\left({f}{^{\prime}}\right)}^{2}=0.$$

In Eqs. (13) and (14), the term $P$ refers to the pressure. This term can be omitted from both equations by assuming the differentiation of Eq. (14) with respect to $\eta$ to reach at the possible comparison. The resulting equations are given below.

16 $${f}^{iv}+\frac{2}{\eta +K}{f}^{{^{\prime}}{^{\prime}}{^{\prime}}}-\frac{1}{{\left(\eta +K\right)}^{2}}{f}^{{^{\prime}}{^{\prime}}}+\frac{1}{{\left(\eta +K\right)}^{3}}{f}{^{\prime}}+\frac{{v}_{f}}{{v}_{hnf}}\left[\frac{K}{\eta +K}\left(f{f}^{{^{\prime}}{^{\prime}}{^{\prime}}}-{f}{^{\prime}}{f}^{{^{\prime}}{^{\prime}}}\right)+\frac{K}{{\left(\eta +K\right)}^{2}}\left(f{f}^{{^{\prime}}{^{\prime}}}-{{f}{^{\prime}}}^{2}\right)-\frac{K}{{\left(\eta +K\right)}^{3}}f{f}{^{\prime}}-{M}^{2}\frac{{\sigma }_{hnf}}{{\sigma }_{f}}\frac{{\rho }_{f}}{{\rho }_{hnf}}({f}^{{^{\prime}}{^{\prime}}}+\frac{1}{\eta +K}{f}{^{\prime}})\right]=0,$$

17 $$\frac{1}{Pr}\frac{{k}_{hnf}}{{k}_{f}}\frac{{\left(\rho {C}_{p}\right)}_{f}}{{\left(\rho {C}_{p}\right)}_{nf}}(1+\frac{4}{3}Rd)\left(\theta {^{\prime}}{^{\prime}}+\frac{1}{\eta +K}\theta {^{\prime}}\right)+\frac{K}{\eta +K}f{\theta }{^{\prime}}+{\frac{{\mu }_{hnf}}{{\mu }_{f}}\frac{{\left(\rho {C}_{p}\right)}_{f}}{{\left(\rho {C}_{p}\right)}_{hnf}}E}_{c}{\left({f}^{{^{\prime}}{^{\prime}}}-\frac{{f}{^{\prime}}}{\eta +K}\right)}^{2}+{{M}^{2}E}_{c}\frac{{\left(\rho {C}_{p}\right)}_{f}}{{\left(\rho {C}_{p}\right)}_{hnf}}\frac{{\sigma }_{nf}}{{\sigma }_{f}}{\left({f}{^{\prime}}\right)}^{2}=0.$$

The associated dimensionless conditions are shown below.

18 $$\left.\begin{array}{c}f\left(0\right)=0, {f}{^{\prime}}\left(0\right)=\lambda =\frac{c}{a}, \theta \left(\eta \right)=1,\\ {f}{^{\prime}}\left(\eta \right)=1, {f}^{{^{\prime}}{^{\prime}}}\left(\eta \right)=0, \theta \left(\eta \right)=0\,\, as\,\, \eta \to \infty .\end{array}\right\}.$$

In terms of PHF, just $\theta$ is replaced with $g$ in Eq. (17) although the entire equation remains unchanged^40^. However, the boundary conditions are modified which are presented hereunder.

19 $${g}{^{\prime}}\left(0\right)=-1, g\left(\eta \right)=0 \,\,as\,\, \eta \to \infty$$

The parameters appearing in Eqs. (16–19) are defined below

$$\lambda =\frac{c}{a},K=R\sqrt{\frac{a}{{v}_{f}}} , {M}^{2}=\frac{{\sigma }_{f}{{B}_{0}}^{2}}{a{\rho }_{f}}, Pr=\frac{{\nu }_{f}}{{\alpha }_{f}}, {E}_{c}=\frac{{u}^{2}}{{C}_{p}\Delta T}=\frac{{a}^{2}{s}^{2}}{{C}_{p}({T}_{w}-{T}_{\infty })}, Rd=\frac{4{\sigma }^{*}{T}_{\infty }^{3}}{{k}_{f}{k}^{*}}$$

The local Nusselt number $({Nu}_{L})$ and the skin friction coefficient $({C}_{f})$ refers to the properties of engineering interest which may be illustrated as^41^.

20 $${C}_{fr}=\frac{{\tau }_{w}}{{\frac{1}{2}\rho }_{f}{{u}_{e}}^{2}(s)}, {Nu}_{L}=\frac{s{q}_{w}}{{k}_{f}\left({T}_{w}-{T}_{\infty }\right)}.$$

where

21 $${\tau }_{w}={\mu }_{hnf}{\left.\left(\frac{\partial u}{\partial r}-\frac{u}{r+R}\right)\right|}_{r=0}, {q}_{w}=-{k}_{hnf}{\left.\frac{\partial T}{\partial r}\right|}_{r=0}.$$

In above system (20), the term ${\tau }_{w}$ and ${q}_{w}$ refers respectively to the shear stress and heat flux through the surface which are defined above in (21).

Considering the use of Eq. (12) in system (20) leads to the dimensionless structure as stated below.

22 $$\begin{aligned} ~C_{f} {\text{~}} & = \left( {Re_{s} } \right)^{{\frac{1}{2}}} {\text{~}}C_{{fr}} = \frac{{\mu _{{hnf}} }}{{\mu _{f} }}\left\{ {f^{\prime\prime}\left( 0 \right) - \frac{\lambda }{K}} \right\}, \\ Nu & = \left( {Re_{s} } \right)^{{ - 1/2}} {\text{~}}Nu_{L} {\text{~}} = - \frac{{k_{{hnf}} }}{{k_{f} }}\left( {1 + \frac{4}{3}Rd} \right)\theta ^{\prime}\left( 0 \right). \\ \end{aligned}$$

where ${Re}_{s} =\frac{a{s}^{2} }{{\nu }_{f}}$ represent the localized Reynolds number.

## Discussion and results

This section considers the numerical and graphical solutions to the nonlinear dimensionless ODEs (16) and (17) depending on the coupled boundary conditions (18) and (19). The graphical solutions are presented as a consequence of velocity, temperature, skin friction coefficient, pressure and local Nusselt number. The MATLAB built-in package bvp4c has been used to see the impact of diverse flow parameters such as Eckert number $(\mathrm{Ec})$, Prandtl number $(\mathrm{Pr})$, curvature parameter $(\mathrm{K})$, volume fraction of nano particles $({\upphi }_{1}, {\upphi }_{2})$, radiation parameter $(\mathrm{Rd})$, stretching parameter $(\uplambda )$, and Hartmann number $(\mathrm{M})$ on the stated properties of the hybrid nanofluid. The nanoparticles and the base fluid are referring to the thermophysical properties provided in Table 2.

**Table 2:** Thermophysical properties of the base fluid (${C}_{2}{H}_{6}{O}_{2}$) and the nanoparticles ($A{l}_{2}{O}_{3}$) and $Cu$.^24,41^.

Thermophysical properties	($$f)$$ ($${C}_{2}{H}_{6}{O}_{2}$$)	($${s}_{1}$$) ($$A{l}_{2}{O}_{3}$$)	($${s}_{2}$$) Cu
${C}_{p} \,\,({\rm J}/{\rm kgK})$	2430	765	$385$
$\rho \,\,\left( {{\text{kg/m}}^{{{3}}} } \right)$	1115	3970	$8933$
$k\,\, ({\rm W}/{\rm mK})$	0.253	40	$400$
$\sigma \,\,({\rm S}/{\rm m}$)	${1\times 10}^{-7}$	${35\times 10}^{6}$	$59.6\times {10}^{6}$

**Figure 2:**
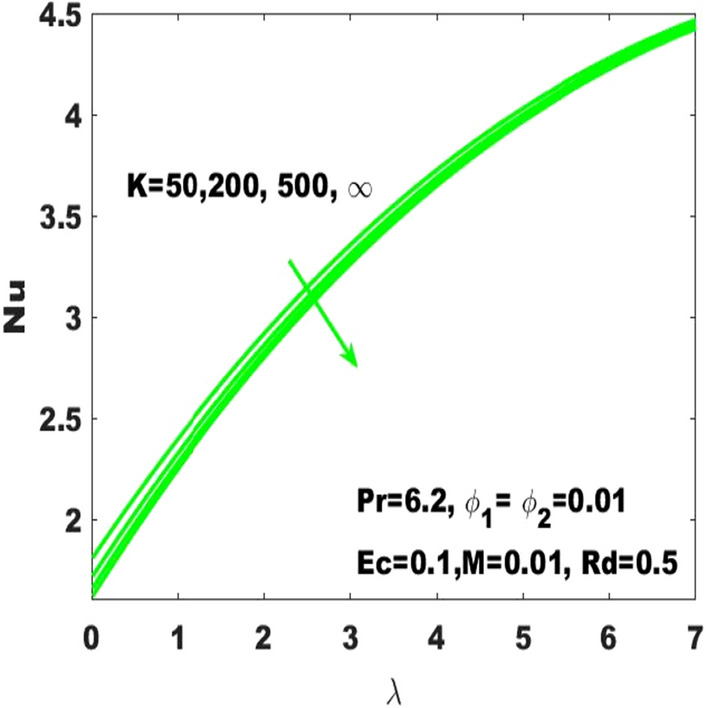
Change in $\mathrm{Nu}$ with $\uplambda$ for diverse values of $\mathrm{K}$.

**Figure 3:**
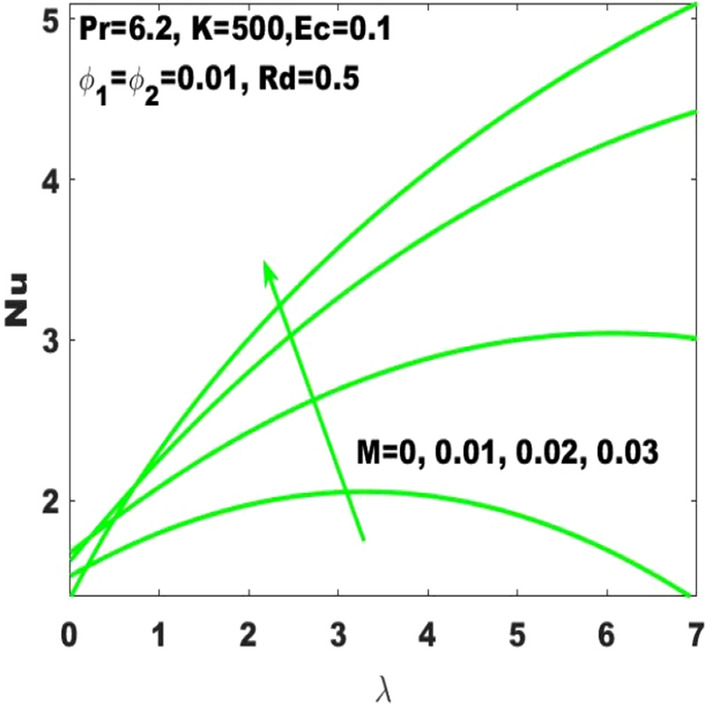
Change in $\mathrm{Nu}$ with $\uplambda$ for diverse values of $\mathrm{M}$.

**Figure 4:**
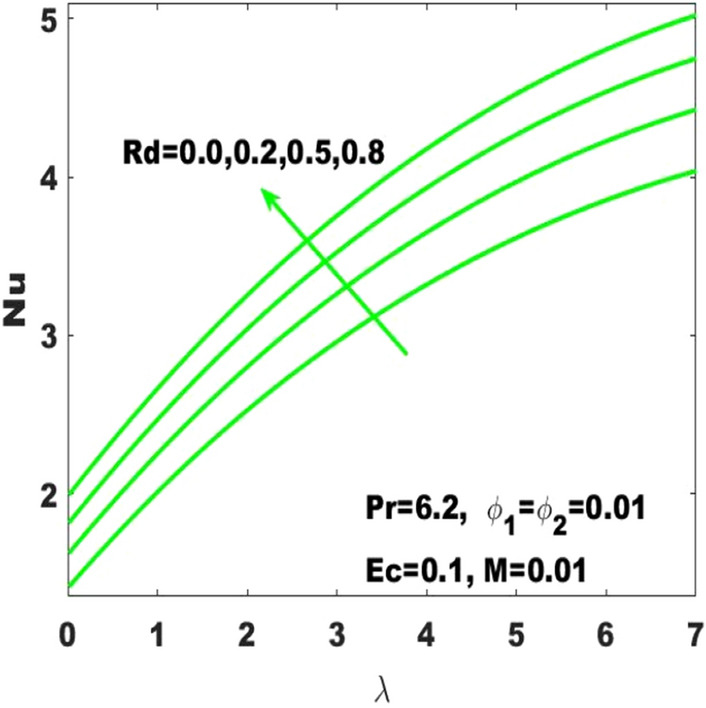
Change in $\mathrm{Nu}$ with $\uplambda$ for diverse values of $\mathrm{Rd}$.

The Nusselt number $(\mathrm{Nu})$ which is called the “rate of heat transfer” is varying individually under the consideration of different irregular values of the curvature parameter, Hartmann number and radiation parameter as respectively displayed in Figs. 2, 3 and 4. From Fig. 2, it is obvious that the rate of heat transfer is moderately sensible to the surface curvature as it slightly decreases with increasing curvature. Finally, one can observe that the Nusselt number is smallest for the maximum values ($\mathrm{K}\to \infty$) of the curvature parameter which declares that the rate of heat transfer is dominant with the consideration of flat surface as the curved surface turns into the flat surface with $\mathrm{K}\to \infty.$

Moreover, it is obvious that higher surface stretching is more sensitive to the rate of heat transfer.

In Fig. 3, it is noticed that the rate of heat transfer is affected intensively due to the change in Hartmann number $(\mathrm{M})$ as it is enormously enhancing due to the slight boost in $\mathrm{M}$. In the absence of magnetic field, the heat with a minimum rate is transferred. Considering high values of $\mathrm{M}$ slumps the fluid motion due to the magnetic field and thus the heat transfer rate is dominant. In the same way, Nusselt number $(\mathrm{Nu})$ is dominant with higher rate of stretching. In addition, the Nusselt number is highly influenced by the radiation parameter subject to the increasing rate of surface stretching. Definitely the speeding up of the thermal radiation accelerates the rate of heat transfer as seen in Fig. 4. Most of the industrial, electrical, and electronic appliances may not work without the consideration of heat transmission. Therefore, the fluid motion considering the heat transmission is very important to utilize in such types of materials.

**Figure 5:**
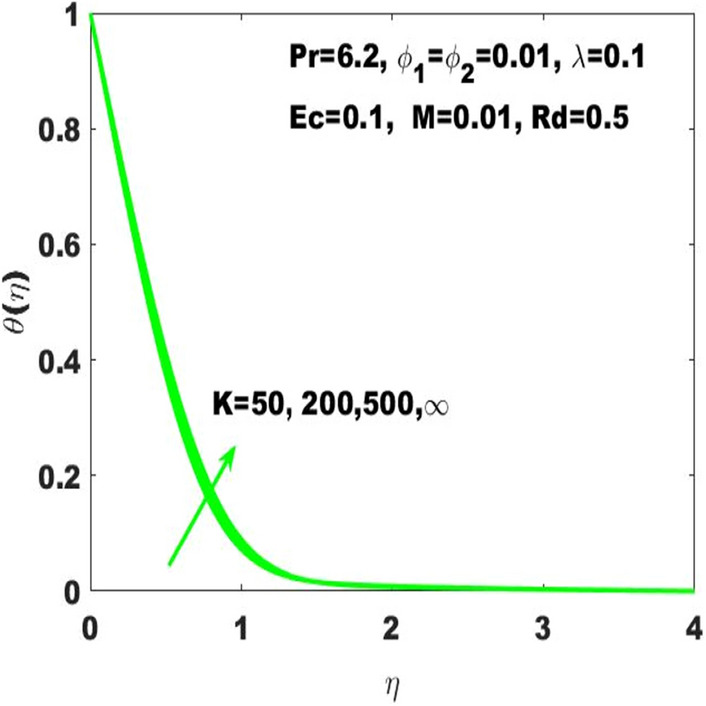
$\uptheta \left(\upeta \right)$ for diverse values of $\mathrm{K}.$

**Figure 6:**
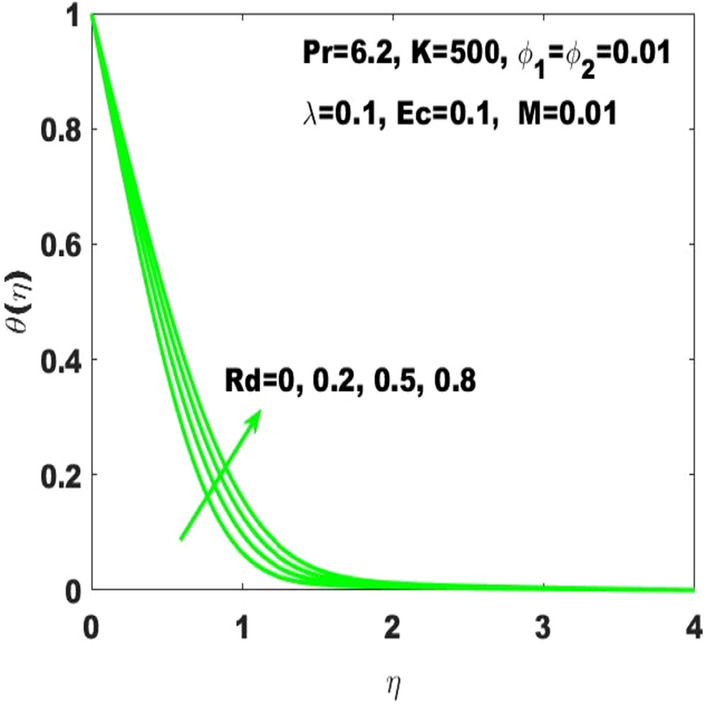
$\uptheta \left(\upeta \right)$ for diverse values of $\mathrm{Rd}.$

**Figure 7:**
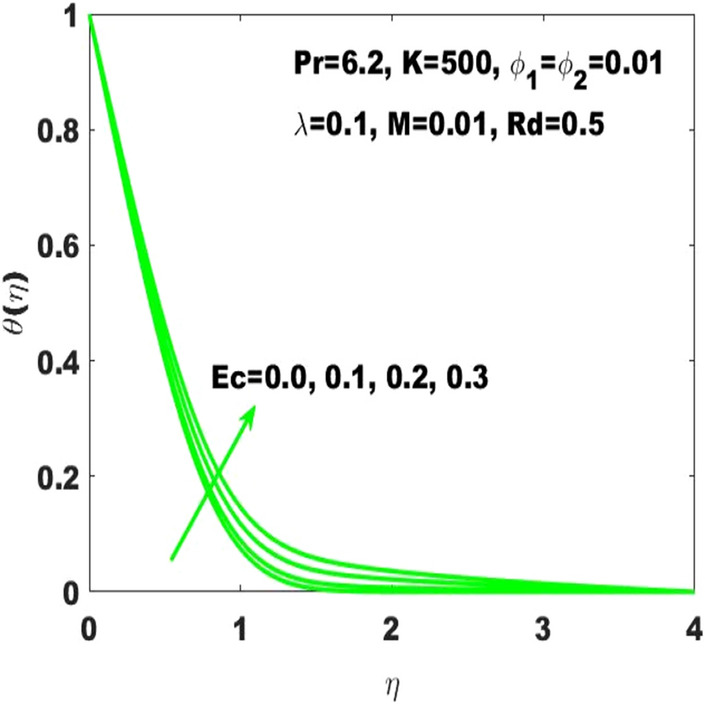
$\uptheta \left(\upeta \right)$ for diverse values of $\mathrm{Ec}.$

The amendments in the fluid temperature profile $\uptheta \left(\upeta \right)$ against the curvature parameter $(\mathrm{K})$ is displayed in Fig. 5. The graphical observation indicates that the building up of curvature supports the fluid temperature to boost it alternatively stated that the plane surface is more effective in boosting of fluid temperature as the curved surface turns into the flat surface with $\mathrm{K}\to \infty$ Additionally, it may be observed that $\uptheta \left(\upeta \right)$ is respectively higher and smaller corresponding to the case of PST and PHF providing the rise in curvature. Figure 6 is supposed to show that how $\uptheta (\upeta )$ is controlled by variation in thermal radiation $\mathrm{Rd}$. It is obvious that the fluid temperature profile has ascending behavior against the accelerating values of the $\mathrm{Rd}$ as the speeding up of thermal radaitions conveys larger heat towards the fluid and thus it causes to strengthen the thickness of thermal boundary layer. Accordingly, the fluid temperature leads to be ascended. It is also important to determine that how the divers values of Eckert number $(\mathrm{Ec})$ lead to the change in fluid temperature $\uptheta \left(\upeta \right)$ assuming the rest parameters at constant values. In this regard, $\uptheta \left(\upeta \right)$ has been considered for four different values of the Eckert number displayed in Fig. 7. Where $\mathrm{Ec}=0$ leads to the minimum temperature as well as the escalation of $\mathrm{Ec}$ drives the temperature towards higher values. Basically, the kinetic energy ratio to the enthalpy of fluid stands for the Eckert number which imports that the escalation of Eckert number will boost the kinetic energy of the fluid and thus the fluid temperature enhances.

**Figure 8:**
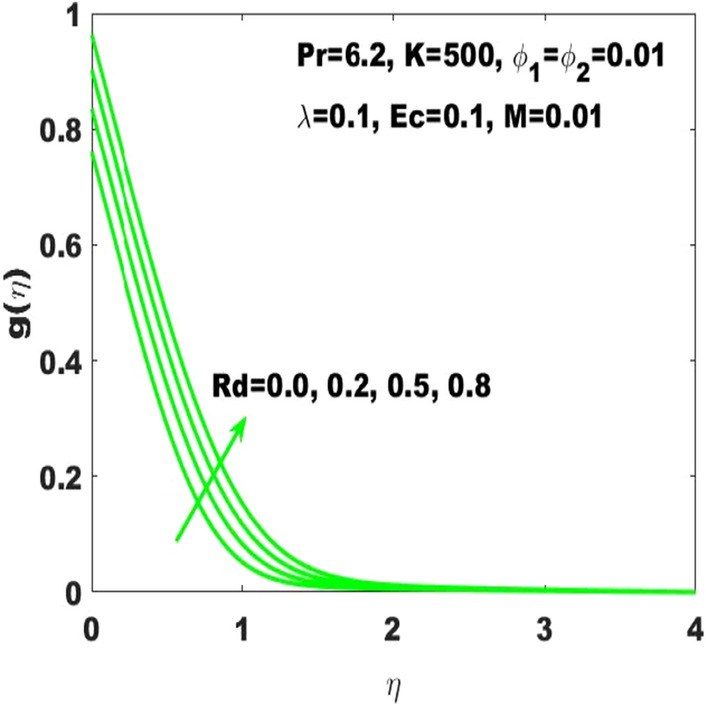
g $\left(\upeta \right)$ for diverse values of $\mathrm{Rd}$.

The graphical result $\mathrm{g}\left(\upeta \right)$ refers to the temperature field based on the PHF, and it is changeable under the consideration of different values of the radiation parameter $(\mathrm{Rd})$ as displayed in Fig. 8. From this figure, it is obvious that $\mathrm{g}\left(\upeta \right)$ has ascending behavior against the accelerating values of $\mathrm{Rd}$ as the speeding up of thermal radaitions conveys larger heat towards the fluid and thus it causes to strengthen the thickness of thermal boundary layer. Accordingly, $\mathrm{g}\left(\upeta \right)$ leads to be ascended.

**Figure 9:**
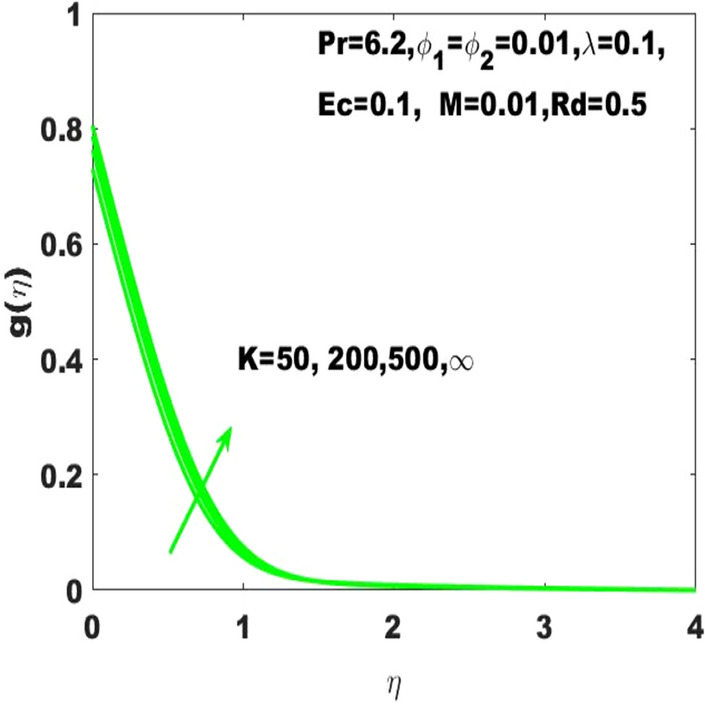
g $\left(\upeta \right)$ for diverse values of $\mathrm{K}.$

In the same way, the changes in the $\mathrm{g}\left(\upeta \right)$ against the curvature parameter $(\mathrm{K})$ is demonstrated in Fig. 9. The graphical observation indicates that the building up of curvature supports the fluid temperature to boost it alternatively stated that the plane surface is more effective in boosting of fluid temperature as the curved surface turns into the flat surface with $\mathrm{K}\to \infty$. The important point is that the fluid temperature is smaller than the temperature of the surface i.e., $\left({\mathrm{T}}_{\mathrm{w}}>{\mathrm{T}}_{\infty }\right)$. Therefore, the distribution of temperature refers to the PST case is relatively smaller than the case of PHF considering the escalation in $\mathrm{Ec}$.

**Figure 10:**
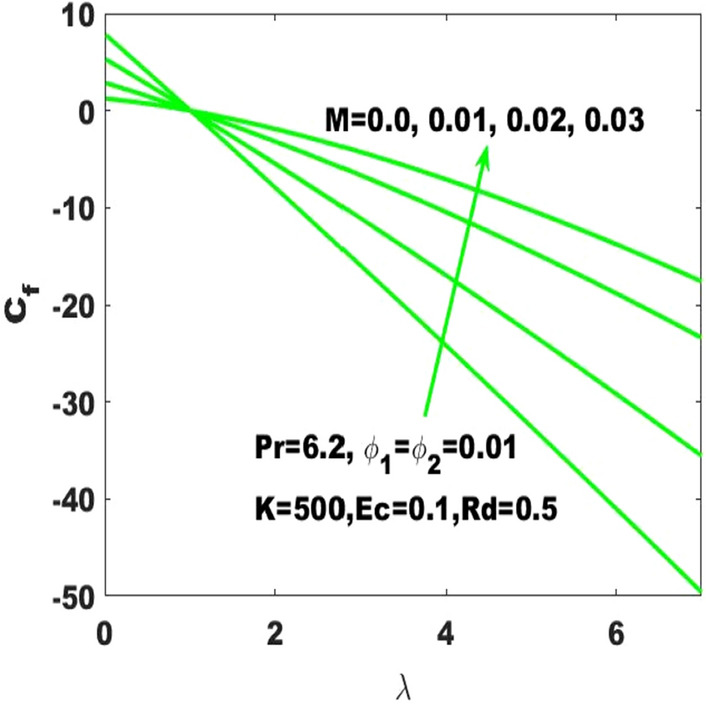
Change in ${\mathrm{C}}_{\mathrm{f}}$ with $\uplambda$ for diverse values of $\mathrm{M}.$

**Figure 11:**
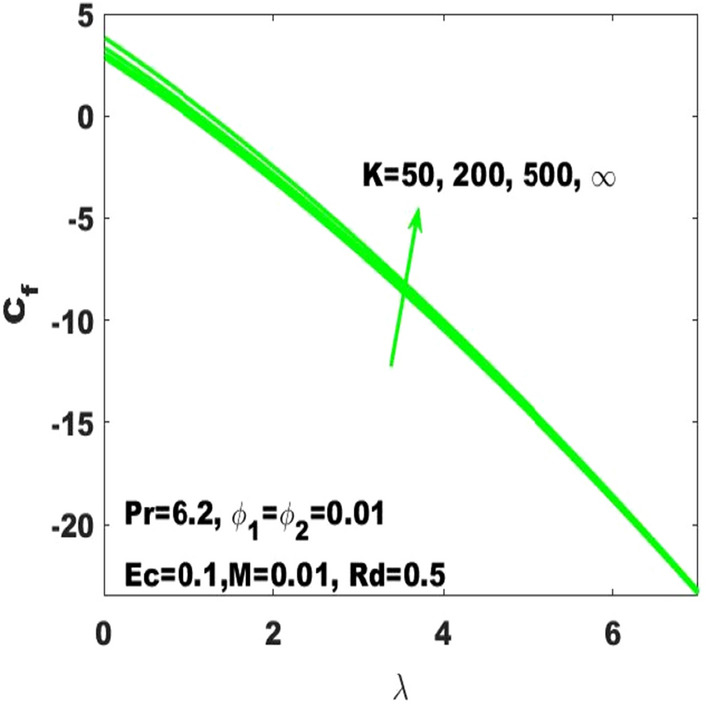
Change in ${\mathrm{C}}_{\mathrm{f}}$ with $\uplambda$ for diverse values of $\mathrm{K}$.

**Figure 12:**
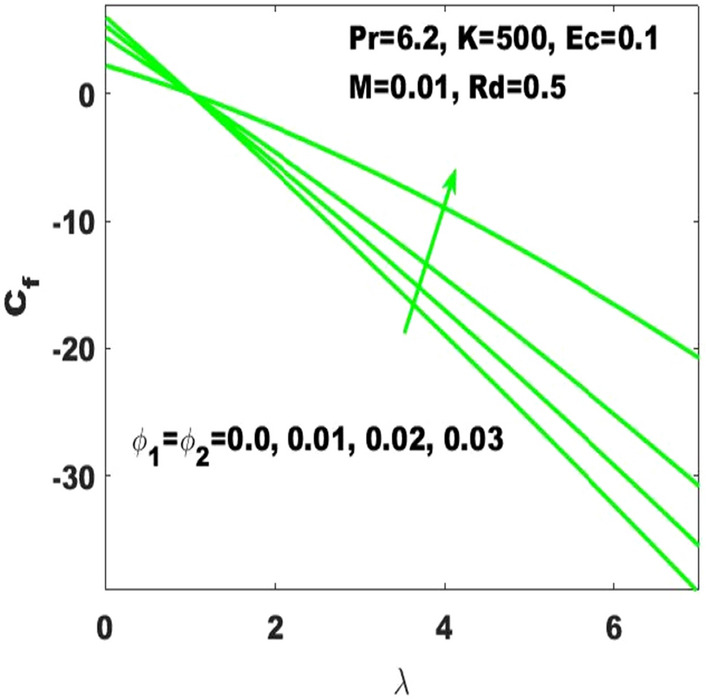
Change in ${\mathrm{C}}_{\mathrm{f}}$ with $\uplambda$ for diverse values of ${\upphi }_{1}$ and ${\upphi }_{2}$.

The assessment of skin friction coefficient (called friction drag) based on the varying values of the Hartmann number ($\mathrm{M}$), curvature parameter $(\mathrm{K})$ and nanoparticles volume fraction $({\upphi }_{1},{\upphi }_{2})$ has been subjected to the stretched surface respectively displayed in Figs. 10, 11 and 12. In Fig. 10, it is noticed that the skin friction coefficient $({\mathrm{C}}_{\mathrm{f}}$) is affected intensively due to the change in Hartmann number $(\mathrm{M})$ as it is immensely enhancing due to the slight boost in $\mathrm{M}$. In the absence of magnetic field, the lowest friction drag is generated. On the other hand, friction drag is ineffective with the higher rate of stretching. From Fig. 11, it is apparent that the friction drag is quite sensible to the surface curvature parameter as it slightly enhances with rising values of curvature paramter. Ultimately, one can declare that the skin friction coefficient is highest for the maximum values ($\mathrm{K}\to \infty$) of the curvature parameter which affirms that the friction drag is dominant with the consideration of flat surface as the curved surface turns into the flat surface with $\mathrm{K}\to \infty$. Additionally, it is noticeable that higher surface stretching controls the skin friction coefficient. It is also evident that ${\mathrm{C}}_{\mathrm{f}}$ is ascending with the high strength of nanoparticles volume fraction $({\upphi }_{1}, {\upphi }_{2})$ as displayed in Fig. 12. The distribution of excess number of nanoparticles leads to the probability to enhance the friction among the fluid layers and resulting to enhance the resistance. Alternatively, it can be stated that the friction drag becomes more effective with the boosting of nanoparticles concentrations. Therefore, the skin friction coefficient refers to the hybrid nanofluid must be relatively larger than the nanofluids.

**Figure 13:**
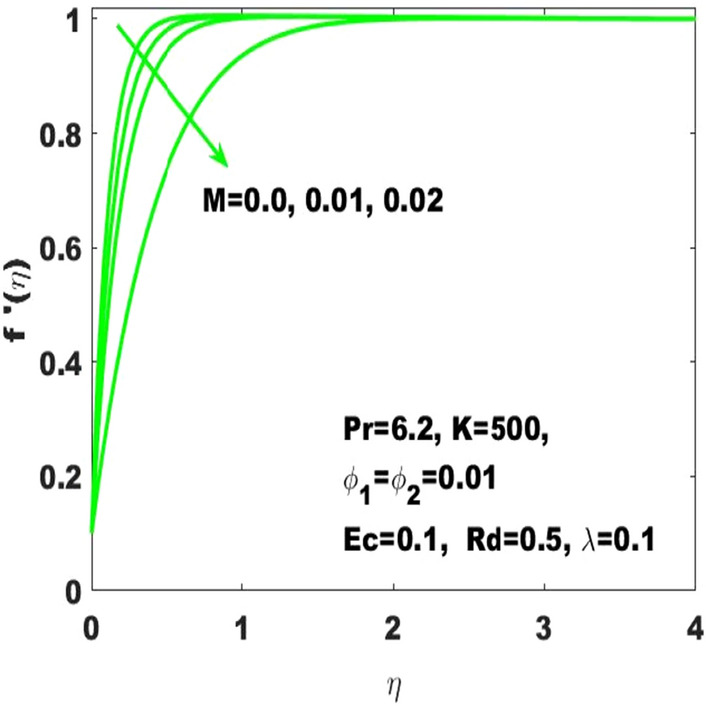
${\mathrm{f}}^{\mathrm{^{\prime}}}\left(\upeta \right)$ for the diverse values of $\mathrm{M}.$

**Figure 14:**
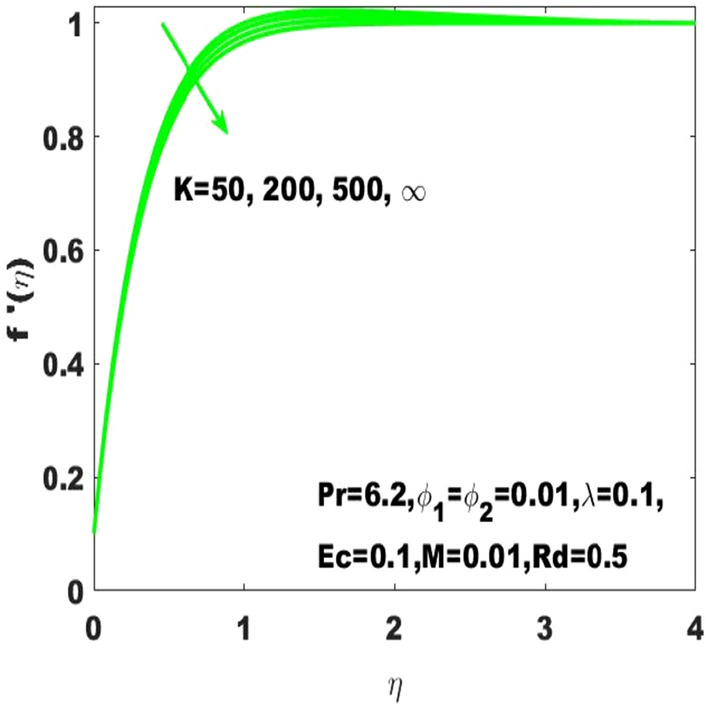
${\mathrm{f}}^{\mathrm{^{\prime}}}\left(\upeta \right)$ for the diverse values of $\mathrm{K}$.

The graphical results ${\mathrm{f}}^{\mathrm{^{\prime}}}\left(\upeta \right)$ describes the velocity field based on the consideration of different values of Hartmann number $(\mathrm{M})$ and the curvature parameter $(\mathrm{K})$ which are respectively displayed in Figs. 13 and 14. From these figures, it is obvious that ${\mathrm{f}}^{\mathrm{^{\prime}}}\left(\upeta \right)$ has descending behavior against the accelerating values of Hartmann number and curvature parameter $(\mathrm{K})$. Considering high values of $\mathrm{M}$ slumps the fluid velocity due to the magnetic field and thus the boundary layer thickness decays. In this way, the bulk motion of the electrically conductive nanofluid is limited. The experimental work in hydromagnetic flow has been associated with the applied magnetic field used to control the boundary layer thickness. This clarifies that the experimental work in hydromagnetic flow supports our theoretical suppositions. Moreover, the accelerating values of $\mathrm{K}$ leads to the smallest dimentionless curvature of the curved surface due to which the friction darg increase which consequently declines the fluid velocity.

**Figure 15:**
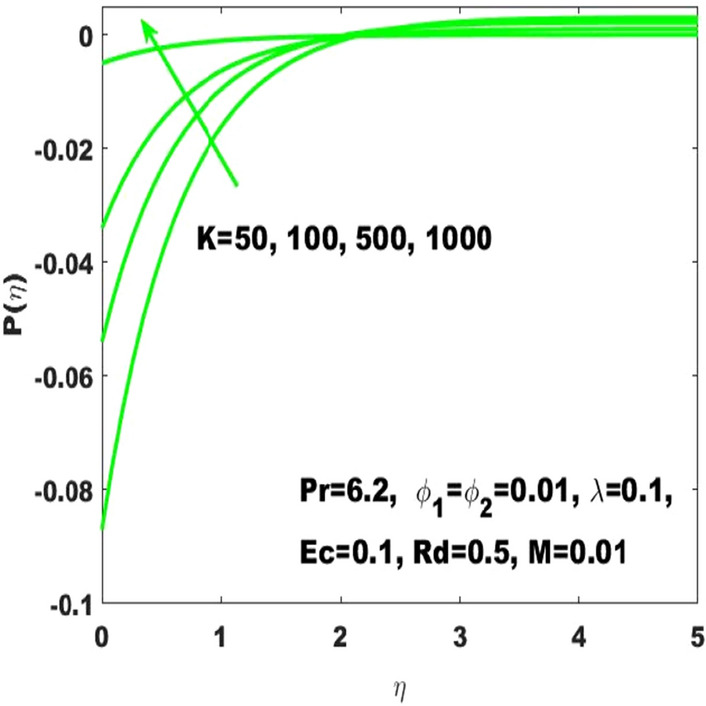
$\mathrm{P}\left(\upeta \right)$ for the diverse values of $\mathrm{K}.$

**Figure 16:**
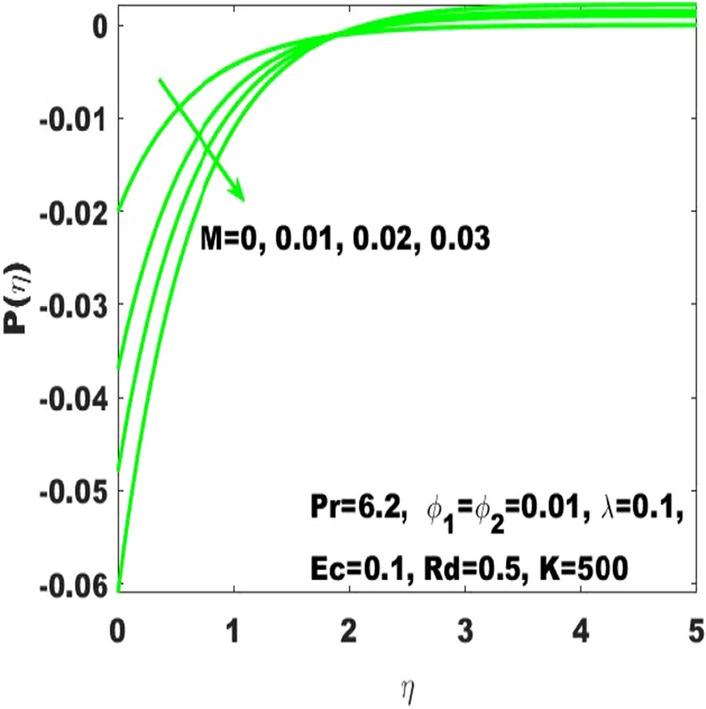
$\mathrm{P}\left(\upeta \right)$ for the diverse values of $\mathrm{M}.$

In the study of curved surface, it is important to consider the impact of a pressure $\mathrm{P}\left(\upeta \right)$. Therefore, Figs. 15 and 16 have been respectively plotted to measure the pressure variations subject to the influence of $\mathrm{K}$ and $\mathrm{M}$. It is determined that the magnitude of $\mathrm{P}\left(\upeta \right)$ enhances with the increase of curvature $\mathrm{K}$ but this effect is limited to the boundary layer. The use of high values of $\mathrm{K}$ like $\mathrm{K}=1000$ leads to the higher values of $\mathrm{P}\left(\upeta \right)$ inside the boundary layer, and thus the highest values of curvature like $\mathrm{K}\to \infty$, (the situation transforming the curved surface into the classical problem of the plane level surface) corresponds to the zero change in pressure both inside and outside the boundary layer. This shows that the pressure variation is very significant considering the curved surface and thus it can’t be neglected. Moreover, it appears that the magnitude of $\mathrm{P}\left(\upeta \right)$ initially declines with the strength of magnetic field, although after $\left(\upeta =2\right)$ it shows an opposite behavior against increasing values of $\mathrm{M}$ as seen in Fig. 16. Equation (14) clearly indicates that the higher values of M will lead to the decrease in pressure. The behavior of $\mathrm{P}\left(\upeta \right)$ against the accelarating values of K is detected in Fig. 15. It is eident that The clarification behind this action is that $\mathrm{P}\left(\upeta \right)$ diversely behaves both outside and inside the boundary layer. No change in pressure is detection for a flat surface in the exterior or interior of the boundary layer, although the existence of curvature directly influenced the pressure specifically in the interior of the boundary layer. Moreover, the diverse values of the parameters and the results of the study are in good agreement with the work of Sajid et al.^31^. Additionally, the validation of numerical result of ${\mathrm{C}}_{\mathrm{f}}$ is presented in Table 3 where it is observed that the solutions obtained in this article are in appreciable accuracy with the existing literature. The numerical results of the skin friction coefficient and the Nusselt number is respectively presented in Tables 4 and 5 alternatively characterizes the friction drag and heat transport rate subject to the diverse values of the parameters. It can be noted from Table 4 that the increase in the radius of curvature leads to a decrease in the skin friction coefficient whereas the increase in the volume fractions $({\upphi }_{1}, {\upphi }_{2})$ enhances the friction drag. Meanwhile, increase in the radius of curvature decreases the Nusselt number as well as shown in Table 5 and also the increase in the Eckert number has the same effect. The higher values of radiation parameter increases the Nusselt number.

**Table 3:** Validation of numerical result of ${\mathrm{C}}_{\mathrm{f}}$ with the published work of Sajid et al.^31^ for the variation of curvature $\mathrm{K}.$

K	Present results	Sajid et al.^42^
5	0.75763	0.75763
10	0.87349	0.87349
20	0.93561	0.93561
30	0.95686	0.95686
40	0.96759	0.96759
50	0.97405	0.97405
100	0.98704	0.98704
200	0.99356	0.99356
1000	0.99880	0.99880

**Table 4:** Behavior of skin friction coefficient (${\mathrm{C}}_{\mathrm{f}}$) under diverse values of $\mathrm{K}, {\upphi }_{1}$ and ${\upphi }_{2}$ when Pr = 6.2, $\uplambda$ =1, Ec = 0.1, M = 0.01 and Rd = 0.5.

$\mathrm{K}$	${\upphi }_{1}$	${\upphi }_{2}$	${\mathrm{C}}_{\mathrm{f}}$
50	0.01	0.0	1.9025
100			1.8503
200			1.8243
300	0.00		1.8003
	0.005		1.2437
	0.01		2.2519
	0.02	0.0	2.9031
		0.005	1.2351
		0.01	2.22032

**Table 5:** Heat transport behavior ($\mathrm{Nu}$) under diverse values of $\mathrm{K},\mathrm{ R}$ and $\mathrm{Ec}$ when ${\upphi }_{2}={\upphi }_{1}$ = 0.01, Pr = 6.2, $\uplambda$ =1, and M = 0.01.

$\mathrm{K}$	$\mathrm{R}$	$\mathrm{Ec}$	$\mathrm{Nu}$
50	0.5	0.1	3.0517
100			1.9170
200			1.9048
300	0.0		1.2501
	0.5		1.4098
	1.0		1.9048
	1.5	0.0	2.1270
		0.05	1.9673
		0.1	1.8422
		0.15	1.7797

## Conclusion

The existing work was done to recognize the influence of Joule heating, thermal radiation, and viscous dissipation over a two-dimensional hydromagnetic flow of a viscous hybrid nanofluid across a stretched curved. The basic PDEs have been transformed into dimensionless ODEs using some specified non-dimensional transformations. The MATLAB built-in package bvp4c has been considered to find the numerical solution to the consequential equations. The diverse values of the dimensionless parameters are used to find the numerical solution which describes the flow characteristics and presents a physical insight of the current work. The remarkable observations taken from various graphical outcomes in terms of heat transport, temperature, pressure, velocity, and friction drag. These remarks states that

- The rate of heat transfer decreases with increasing curvature as well as it is enhancing due to the boost in $M$ and $Rd$.
- The fluid temperature profile $\left(\theta \left(\eta \right)\right)$ has ascending behavior against the accelerating values of the $Rd, K$ and $Ec$.
- The fluid temperature profile $\left(g\left(\eta \right)\right)$ has ascending behavior against the accelerating values of $Rd$ and $K$.
- The skin friction coefficient $({C}_{f}$) is enhancing due to the slight boost in $M, K$ and $({\phi }_{1}, {\phi }_{2})$.
- The ${f}{^{\prime}}\left(\eta \right)$ has descending behavior against the accelerating values of $M$ and $K$.
- The magnitude of the pressure $\mathrm{P}\left(\upeta \right)$ enhances with the increase of $K$ inside the boundary layer.

## Data Availability

The datasets used during the current study are available from the corresponding author on reasonable request.

## List of symbols

${\mu }_{f}, {\mu }_{s}$: Nano particles and base liquid dynamic viscosity [${\rm kg{m}}^{-1}{\rm s}^{-1}$] $\left({\rm kg{m}}^{-1}{\rm s}^{-1}\right)$
${{\rho }_{s}, \rho }_{f}$: Nano particles and base liquid density $\left({\rm kg}{\rm m}^{-3}\right)$
${k}_{f}, {k}_{s}$: Base liquid and nano particles thermal conductivity $\left({\rm W}{\rm m}^{-1}{\rm K}^{-1}\right)$
${\nu }_{s},{\nu }_{f}$: Nanoparticles and base liquid kinematic viscosity $\left({\rm m}^{2}/{\rm s}\right)$
${{({C}_{p})}_{f}, ({C}_{p})}_{s}$: Base liquid and nanoparticles specific heat capacity $[{\mathrm{ML}}^{2}/{\mathrm{T}}^{2}\mathrm{K}]$ $\left({\rm Jk}{\rm g}^{-1}{\rm K}^{-1}\right)$
${\sigma }_{f}, {\sigma }_{s}$: Base liquid and nano particles electrical conductivity $\left({\Omega }^{-1}{m}^{-1}\right)$
${\alpha }_{f}, {\alpha }_{s}$: Base liquid and nanoparticles thermal diffusivity $\left({\rm m}^{2}/{\rm s}\right)$
$p$: Pressure of nanofluid $\left({\rm N}/{\rm m}^{2}\right)$
$f$ and $\psi$: Dimensionless stream function $\left(-\right)$
${C}_{f}$: Skin friction coefficient $\left(-\right)$
$${Re}_{x}$$: Reynolds number $$(-)$$
$$\varphi$$: Nanoparticle’s concentration $$\left(-\right)$$
${B}_{0}$: Magnetic field strength $\left(\mathrm{Tesla }\,\,{\rm N}/{\rm Am}^{2}\right)$
${T}_{w}, {T}_{\infty }$: Surface and ambient temperatures $\left(K\right)$
$$M$$: Hartmann number $$\left(-\right)$$
$$Ec$$: Eckert number $$\left(-\right)$$
$${\nu }_{w}$$: Suction velocity $$\left({\rm m}{\rm s}^{-1}\right)$$
Rd: Radiation parameter $\left(-\right)$
$${\tau }_{w}$$: Shear stress $$\left({\rm Pa}\,\, {\rm or}\,\, {\rm N}/{\rm m}^{2}\right)$$
$K$: Radius of curvature $\left({\rm m}\right)$
${\mu }_{hnf}$: Hybrid nanofluid dynamic viscosity $\left({\rm kg}{\rm m}^{-1}{\rm s}^{-1}\right)$
${\rho }_{hnf}$: Hybrid nanofluids density $\left({\rm kg}{\rm m}^{-3}\right)$
${k}_{hnf}$: Hybrid nanofluids thermal conductivity $\left({\rm W}{\rm m}^{-1}{\rm K}^{-1}\right)$
${\nu }_{hnf}$: Hybrid nanofluid kinematic viscosity $\left({\rm m}^{2}/{\rm s}\right)$
$\left( {C_{p} } \right)_{{hnf}}$: Hybrid nanofluids heat capacity $\left( {{\rm jkg}^{{ - 1}} {\rm K}^{{ - 1}} } \right)$
${\sigma }_{hnf}$: Hybrid nanofluids electrical conductivity $\left(\left({\Omega }^{-1}{\rm m}^{-1}\right)\right)$
${\alpha }_{hnf}$: Hybrid nanofluids thermal diffusivity $\left({\rm s} {\rm m}^{2}\right)$
$$\theta$$: Dimensionless temperature $$\left(-\right)$$
$\eta$: Similarity space variable $\left(-\right)$
$${Nu}_{x}$$: Nusselt’s Number $$\left({\rm hd}{\rm k}^{-1}\right)$$
$T$: Temperature of the fluid $\left(K\right)$
$$a$$: Stretching constant $$\left(-\right)$$
$u, v$: Velocity components in $x, y$ directions respectively $\left({\rm ms}^{-1}\right)$
$$\lambda$$: Surface stretching $$\left(-\right)$$
${k}^{*}$: Coefficient of mean absorption $\left({\rm cm}^{-1}\right)$
$$Pr$$: Prandtl number $$\left(-\right)$$
$${f}{^{\prime}}$$: Dimensionless velocity $$\left(-\right)$$
${\sigma }^{*}$: Stefan-Boltzmann constant $\left(\mathrm{W}/{m}^{2}\right)$
$${q}_{w}$$: Heat flux $$\left(\mathrm{W}/{m}^{2}{K}^{4}\right)$$
${q}_{r}$: Radiative heat flux $\left(\mathrm{W}/{m}^{2}\right)$
$$P$$: Dimensionless pressure $$\left(\mathrm{N}/{m}^{2}\right)$$
